# Appraising Virtual Technologies’ Impact on Older Citizens’ Mental Health—A Comparative between 360° Video and Virtual Reality

**DOI:** 10.3390/ijerph191811250

**Published:** 2022-09-07

**Authors:** Lucas Kupczik, William Farrelly, Shane Wilson

**Affiliations:** Department of Computing, Atlantic Technological University Donegal, Letterkenny Campus, Port Road, F92 FC93 Letterkenny, Ireland

**Keywords:** virtual reality, 360° videos, immersive technology, older people, healthcare, psychological health, nature, outdoors, digital health, sensor technology

## Abstract

Aging populations across the world are facing a number of challenges in the context of health and healthcare. These challenges are driven by the aging process and the illnesses associated with aging. Healthcare for older people has become a point of concern with most health organizations, and this is particularly the case with palliative care. In this instance, the movement of the patient may be restricted to a room with no or limited access to the outdoors. This research focuses on the active integration of immersive technologies with healthcare. By addressing the problem of providing patients with the experience of being present in an outdoors space, the associated psychological and physiological benefits can be identified. In this mixed methods research paper, the impact of a crossover study to discern technology preferences in relation to immersive technologies among a sample of older people is reported. In addition, the study highlights factors that contribute to a meaningful immersive experience that can improve psychological and physiological wellbeing. The study identifies that there are two significant categorical aspects that contribute to such immersive experiences, technological aspects (including, for example, the weight of headsets, visual impairment, pixelation, and gamification) and emotive aspects (for example, joy, anger, and fear). The study suggests that older people prefer immersive Virtual Reality (VR) environments rather than 360 video experiences. This can be attributed to the greater flexibility in the provision of interactivity in virtual reality systems.

## 1. Introduction

The size of the older population is increasing in Europe as an outcome of advances in medicine, improved healthcare, sanitation, and increased life expectancy [1,2,3]. Providing cost-effective support for the aging population is a key objective for governments [4,5], as aging is related to a number of common conditions, such as reduced mobility [6], dementia [7,8,9,10], Alzheimer [10,11,12], and other physiological or psychological problems [13,14,15]. These lead to reduced independence for the individuals, resulting in an increased number of older people being admitted to nursing homes and requiring assistance for living [16,17]. This leads to a deterioration in both mental and physical health [1,8,14,18,19], resulting in depression and anxiety [1,2,20,21,22,23], loneliness [24,25,26], and sedentarism [8,27].

Immersive technologies, such as virtual reality (VR), augmented reality (AR), and mixed reality (XR), have been demonstrated to be effective tools in the therapeutic treatment of posttraumatic stress disorder [20,28], phobias [29,30], dementia [22,31], loneliness, and stress [12,32,33,34], and such systems have a positive influence on older people’s mental and physical wellbeing.

VR technology has improved dramatically over the last decade, offering better quality and accessible pricing [20,35,36] and providing affordable tools for entertainment and clinical healthcare [32,37,38,39]. With this emerging technology, it is possible to create highly immersive and realistic environments for individuals with reduced mobility [32,37,40]. Contact with nature in virtually created outdoor environments has been used as treatment to recover from mental fatigue and stress [24,33,41,42]. However, several challenges remain, including: the control of simulation sickness during VR experiences [11,14,22,37,39,43]; reduction in induced discomfort [37,43]; assessment of the level of realism necessary to provide an immersive experience for participants; and an evaluation of which technology (VR vs. 360° video) provides the optimal natural outdoor experience.

This paper reviews the current state of the art in terms of the application of immersive technologies with older populations as a means of improving mental health and physical wellbeing. Our research specifically focuses on the challenge of providing older people, with reduced mobility, an opportunity to experience the outdoors using immersive technologies. It also presents the results of a crossover study that compares the degree of immersion provided by a virtual outdoor environment with that of a photo-realistic 360° video, focusing on understanding the technology preferences of older people and on the features of the technologies that evidence choice.

## 2. Background

### 2.1. Older People’s Mental Health

The number of people aged 60 or more has increased in the past 30 years [44], representing 20.8% of the EU’s population nowadays, and is expected to grow to 31.3% by 2100 [45]. According to the World Health Organization (WHO), the number of older people aged 60 or more suffering from mental disorders, such as loneliness and depression, is over 15% and growing [10,24,46,47]. The mental health and wellbeing of older people can be influenced by several factors, such as the social environment, their physical condition, family presence, overcrowding, and the entertainment available daily [14,22,46]. Common illnesses, for example, vulnerability syndrome and dementia, in addition to the physiological restrictions of sedentarism and a lack of contact with nature, can lead to psychological problems, such as anxiety and depression [7,19,23], stress [10,24,48], and the feeling of loneliness [24,25]. In attempts to deal with the common psychological and physiological issues experienced by older citizens, several therapeutic approaches have been trialed to help reduce pain and improve the mental and physical health of older people [4,17,39,49]. These include the integration of nostalgia [12], therapeutics, music, animal assistance, exercising, and access to the outdoors [8,17,22,49]. Access to the outdoors and contact with nature have been shown to improve physiological and psychological wellbeing [1,39,50].

Contact with an open-air environment is one of the most common interventions used by carers for older people suffering from anxiety, depression, and dementia [1,8,14,22,23,33]. Unfortunately, access to outdoor environments, such as gardens, public parks, or remote wilderness locations, can be limited or non-existent for many older people with little or no mobility [51]. For mobility-restricted individuals, virtual reality can ‘fill the gap’ by providing an opportunity to take a virtual walk in the countryside.

### 2.2. Immersive Technologies and Healthcare

Immersive technologies have been growing over the past years in diverse areas [36,52], such as healthcare [53,54], education [55,56], and entertainment [5,37]. Immersive technology operates by merging virtual simulated reality with the physical world [36], using the sense of presence to create a believable experience, simulating the feeling of ‘being there’. ‘Immersion’ is the degree to which a virtual reality experience can stimulate the senses of the user and it is achieved by manipulating a set of high-fidelity audio and visual components [39]. The stimulation of senses, created by immersive environments, is considered a key contributing factor in the achievement of believable experiences [37,38,39]. The management of stress caused by immersion is a key criterion in the evaluation of the suitability of an immersive experience.

The response to immersion can produce a feeling of ‘being somewhere else’ rather than at the current physical location [10,38,39,57]. The response can range from unconscious physiological processes (cerebral, cardiac, and breathing) to deliberate behaviors [38], for example, trying to interact with objects in a virtualized scenario.

While a range of immersive experiences that utilize VR and 360° videos technologies are available, little research has been undertaken to critically evaluate and compare the level of immersion achieved with target audiences. The objective of this research is to differentiate between participants’ experiences with each of the technologies and to discuss the advantages and disadvantages of using both technologies with older people.

#### 2.2.1. Immersive 360° Video

The 360° videos take the user into the digital world where the user becomes part of the video [58]. A 360° video permits a user to become immersed in a digital representation of a real-world scenario (going for a walk in the country). Formatted as a playable video, where the user has a 360° view of the real world accessed through a VR headset, the user has otherwise limited control (the ability to stop, restart, pause, and choose a route at particular junctures). Moreover, 360° videos provide a quick and less expensive way to develop immersive virtual environments for older people [38]. The 360° videos demonstrate a capacity to capture the user’s sense of presence by using video reproduction techniques, for example, positioning the camera at an adaptive height to suit the viewpoint of the user (user may be seated or standing up, for example), using surround sound to captivate attention, and selecting the greatest image resolution possible [38].

However, the biggest disadvantage of a 360° video is the poor homogenization of the pixels due to the compression technology [38,58], although 360° cameras have evolved over the years, minimizing this issue [59], and the lack of interactions and freedom to engage with the environment [38,60]. However, layered VR/AR can create a more varied experience.

#### 2.2.2. Virtual Reality (VR)

Virtual reality creates a digital virtual space in which digital objects can be manipulated and offers users significantly higher levels of interaction than 360° videos. Unlike 360° video experiences where the user passively follows a pre-recorded path, simulated VR experiences can provide users with the freedom to explore and interact with virtual objects within the environment [38].

The ability to explore a virtual space and interact with objects within VR experiences has been shown to increase the level of immersion for users [38,39,61]. Mol (2019) [39] reported that older users were able to enjoy VR experiences more when they were able to independently explore the virtual environment by themselves instead of following predefined routes.

However, while providing users with the ability to freely explore an environment increases the user’s level of immersion and satisfaction, it is also more technically challenging to achieve than a 360° video experience. Poor user experience (UX) design, crowded scenes, and non-sequitur visual design can result in non-immersive experiences for users [10]. Poor design implementation and equipment lead to limitations, such as the weight of the equipment [22,37,38], visual acuity causing dizziness/nausea [14,22,37,39,43], and limited vision [11,20,22].

VR has been used in a healthcare context for older people for several reasons, including the examination of: a change in frailty that causes older people to fall [57,62]; the threat phobia of falling [29]; emotional attachment [12,22,32]; and stress recovery through the use of natural sounds [8,14,32,33,37]. Dulau et al.’s (2019) [37] study developed a game, aimed at the prevention of dementia in older adults in the early stages, called ‘A Day to Remember’. It consists of daily life tasks in a virtual environment, such as turning off the alarm, preparation of a lunch bag, and the entry of a correct PIN, but adds a ‘gamified’ attribute that adapts the interface to make tasks simpler and more enjoyable. His study demonstrates the importance of simplifying the interface to help older users to independently play the game. Dulau et al. also found that virtual experiences can result in more satisfying experiences when compared to common mini-mental state examination (MMSE) tests for healthcare. As demonstrated above, the existing research would suggest that properly designed virtual environments can be orchestrated to provide a ‘lived’ experience for frail older people and positively contribute to healthcare policy.

## 3. Materials and Methods

### 3.1. Participants Selection

People older than 59 years, from two community centers, were prefiltered by center managers to participate in the project. Participants were selected based on their fitness level to ensure that no unnecessary risk was encountered. Trials were conducted under strict healthcare guidelines, and strict adherence to COVID-19 regulations was required by all participants in the study. Researchers were required to test negatively for the COVID-19 virus prior to participation. All research protocols and instruments used were approved by the community center managers.

The research team visited each center in advance of the trials to conduct an information day, to explain the aims of the research and provide potential participants with the opportunity to ask questions and try the VR experiences beforehand. Information sheets and consent forms were provided to those who wished to participate in the trials that followed.

### 3.2. Data Collection Methodology

#### 3.2.1. Questionnaire

On the completion of the trials, participants were asked to complete a questionnaire using pen and paper (Appendix A). The questionnaire addressed the participant’s demographic profile and a series of Likert-scale statements using a seven-level scale (−3 = Strongly disagree or displeased to +3 = Strongly agree or pleased, and 0 = neutral). This approach was guided by Best et al.’s (2021) [61] study, which used a similar questionnaire to collect data about how participants react to immersive experiences. The questionnaire also incorporated an open-ended question that allowed participants to express thoughts and justify answers.

#### 3.2.2. Video Observation

Consenting participants were recorded using a mobile device during the trials so that the authors could identify any significant trends from verbal feedback or physiological responses to the experiences. Those recordings constitute observation where researchers identify relevant reactions and categorize them accordingly.

### 3.3. Intervention Implementation Methodology

#### 3.3.1. Equipment

The virtual reality headset used was the HTC Vive headset kit connected to a computer capable of running VR experiences. The headset does not include built-in speakers; therefore, headphones were used for hearing. A swivel chair was used for participants to be able to rotate around while seated, allowing them to maximize the experiences.

#### 3.3.2. Information Day

Information sessions enabled participants to make an informed decision, reducing the potential impact of the novelty of VR in participants’ responses. In this phase, participants were given the opportunity to familiarize themselves with both VR and 360° video experiences for a short time in a specific scene where they could learn the basics of the technology and know what to expect.

#### 3.3.3. Trials

After the information sessions, those participants who agreed to participate in the study further and who had completed consent forms were invited to scheduled trials. At the beginning of the trial, each participant was again asked to confirm that they were in good health, had completed the consent form, and were happy to proceed with the trial.

During the trials, each participant experienced two distinct virtual outdoor experiences. The order in which these were presented to participants was randomized during the trials to avoid selection or accidental bias by participants. A short break between experiences allowed participants to rest and communicate any immediate thoughts or concerns about their experience before proceeding to the second experience.

**Figure 1:**
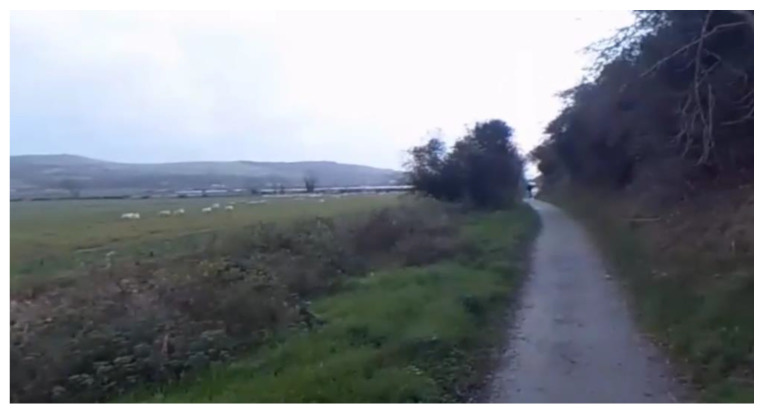
The 360° video experience image representation of video from user’s perspective.

*Experience A* consisted of a 360° video recording of an outdoor walk along a path through a wildfowl sanctuary in Co Donegal, Ireland. The total duration of the video was approximately five minutes, during which participants could explore the environment by looking around the scene (see Figure 1 below) using a VR headset. Participants could not deviate from the route taken during recording but could pause, advance, control volume, or restart the playback of the recording.

The video was recorded by two filmmakers walking down a path surrounded by flora, a river, and sheep in the field. The audible sounds of the video consist of the wind blowing the vegetation and the rain around the camera, and the filmmakers talking. Video of the experience is available on cutt.ly/YT_360Video_Experience [63].

**Figure 2:**
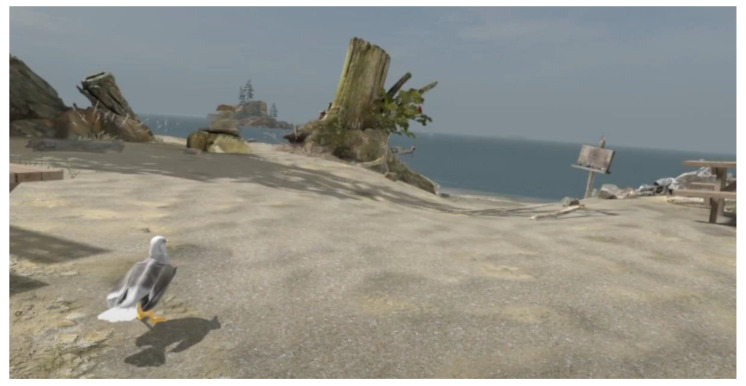
Driftwood beach environment in virtual reality perspective.

Instead of creating an outdoor experience from scratch, for **experience B**, the authors decided to utilize the freely available ‘Driftwood’ simulation (Figure 2), developed by HTC Creative labs [64] and met the requirements for an outdoor environment running on low-cost VR equipment. The simulation also allowed participants to explore both woodland and beach environments in addition to the opportunity to interact with objects in the scene, such as rocks, pebbles, and shells.

Experience B was more complex due to advanced mechanics of interaction and exploration; hence, participants were given 4 min to learn the basics with guidance and 5 more minutes to enjoy the VR experience unguided. Assistance with the navigation and interaction with environment was provided when participants requested.

In the scene, the participants could find animals and admire the horizon with the sea and mountains. The audible sounds of the environment include the waves, minor sound effects from objects (button pressed or dropping objects), seagulls calling, and relaxing music in the background. Video of VR experience is available on cutt.ly/YT_VR_Experience [65].

In both experiences, participants were encouraged to verbalize their thoughts on the experience. All but one of the participants were recorded on video.

### 3.4. Data Analysis Method

#### 3.4.1. Quantitative Analysis

*Phase 1* involved analyzing statistical data (using SPSS) from the questionnaire, focusing on discovering how older participants react to each experience, and on the collection of feedback about: the sense of realism (fidelity of virtual environment in comparison to real world, including graphics, motion, and interactions); the sense of presence associated with the virtual world; awareness of the real world; the degree of captivation associated with the experience; and technology preference. To find the average immersion score and compare both experiences, the mean scores for each question were summed and divided by the number of questions. The survey repeated the content from some questions in an alternative style to reduce bias and ensure validity of the answers.

#### 3.4.2. Qualitative Analysis

*Phase 2* involved categorizing data from qualitative observation of recordings and open-ended questionnaire, which included: positive or negative psychological effects; physical reactions; feedback; issues found or reported during experience; sense of realism; features that caught the participants attention (animals, vegetation, interactions, water, colors); and preference between VR and 360° videos. The reactions were collected through an analysis of visual reactions, such as body movements and actions, facial expressions, and communicative feedback during the experiences.

To compensate for the limitation of the questionnaire to address ‘richness’, a decision was taken to categorize the qualitative data by: psychological effects of technology; issues found; and the immersive features highlighted by participants during trials.

## 4. Results

### 4.1. Analysis

A total of 20 participants (*N* = 20), 3 males and 17 females, tried both the VR and 360° experiences, reviewed it, and answered the questionnaire. The participants were classified into age groups (59−, 60–64, 65–74, 75–84, and 85+). Of the sample population, 35% of the participants were in the 65–74-years age group and 20% of the participants were aged 85 or older, representing a key demographic for this research.

Table 1 below describes the mean values and the significance of each technology for every question of the questionnaire.

Even though the 360° video was recorded in the real world, the table above demonstrates that participants perceived that the VR achieved greater levels of realism and presence than the 360° video experience. The average immersion score indicates that the VR had a higher acceptance rate compared to the 360° video and this is supported by question 21, where 85% of the participants preferred the VR experience. The data suggest that this is because of the freedom to interact with the environment provided by the VR. Previous studies [38,59] have detailed the limitations of 360° videos in terms of interaction and how the ability to explore the virtual space and interact with virtual objects significantly increases the level of immersion for participants in VR experiences.

**Figure 3:**
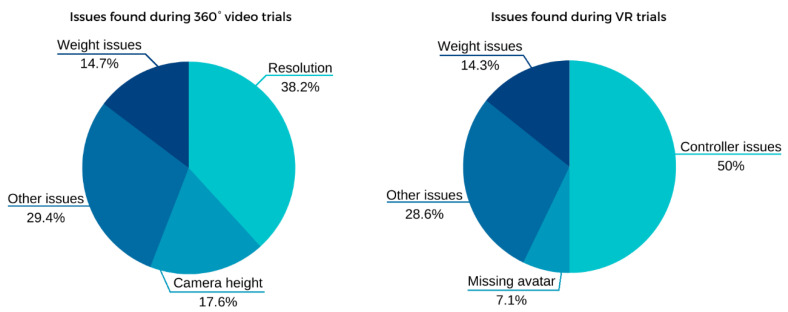
Comparative between issues found during VR and 360° trials.

A careful and detailed analysis of the video recordings of each participant identified several technical and design issues. The authors documented each issue raised by a participant and then placed them into broad categories, as shown in Figure 3.

Many participants (38.2%) reported issues relating to the resolution of the 360° video when it was rendered onto the VR headset. This is a common issue with 360° videos as the technology requires the original video to be captured in an extremely high resolution in order to provide a highly immersive experience. For example, a 4K video has a horizontal resolution of 3840 pixels. As most VR headsets have a field of view of between 90 and 110 degrees, a typical 4K-resolution 360° video would be split into approximately four sections, each with a horizontal resolution of only 1000 pixels. To address the resolution and blurriness issues, the original 360° video should be recorded using a capture resolution of 8K or higher. The processing, storage, and streaming of 8K+ video files which will require several gigabytes presents additional technical challenges.

Another common issue reported by participants was the virtual height of the camera in the 360° videos. During the recording of the 360° video, the camera was held at an approximate height of 2 m off the ground to provide viewers with an unobstructed view of the environment. If the virtual height of the camera significantly differed from the participants’ real-world perspective, we found that participants reported as if they were ‘flying’ through the environment and had little control over the simulation, further reducing the level of immersion.

The most commonly reported issue by participants during the VR experience related to the use of the controller when navigating through the environment or when attempting to interact with objects. During the trials, the participants where verbally instructed on how to navigate and pick up or drop virtual objects. These interactions were also demonstrated to the participants at the beginning of the session. It is clear that more comprehensive guidance on interaction and control mechanics should be provided to participants prior to commencing VR experiences that contain interactive elements.

**Figure 4:**
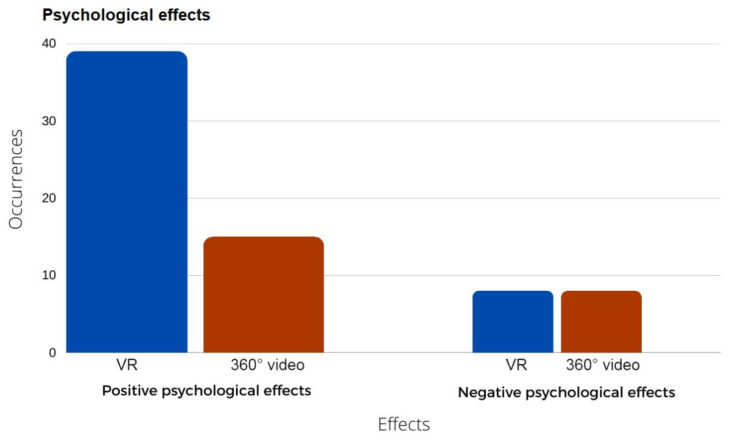
Positive and negative psychological effects occurrences on VR and 360° videos for older people.

The post-trial analysis of the verbal feedback and video observation provided psychological and physiological cues, for example, cues such as ‘*I feel lovely*’ provide a psychological context, while video observations of facial expressions—smiling/laughing and curiosity—and body movements, for example, pointing at a bird with fascination, provide the physiological context. However, negative effects were also reported, such as frustration and being worried; those usually related to the issues above. In total, 39 positive psychological responses for the VR and 15 for the 360° video were identified, while only 8 negative psychological responses were reported for both experiences (Figure 4).

**Figure 5:**
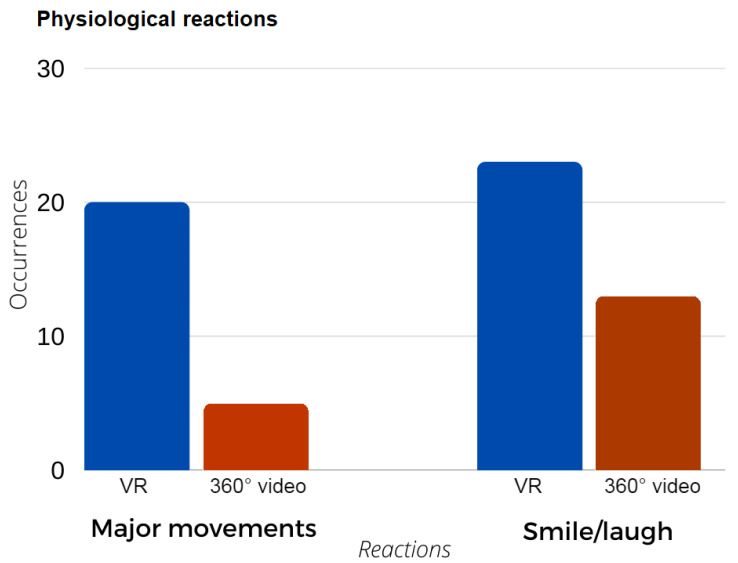
Physiological reactions comparative between VR and 360° videos.

Physical reactions, such as laughing, leg movement, hand gestures, and chair rotation, demonstrate the potential of the technology to incite movement. Figure 5 reflects how the VR incites major movements compared to the 360° video, as it encourages more interaction with the environment.

An independent *t*-test was conducted to compare the level of presence for the VR and 360° video. There was no significant difference in the scores for the 360° video (*M* = 2.20, *SD* = 1.39) and VR (*M* = 2.80, *SD* = 0.41; *t* (22.2) = 1.84, *p* = 0.08, two-tailed). The magnitude of the differences in the means (mean difference = 0.60, 95% CI: −0.07 to 1.27) was medium (Cohen’s d = 0.58).

An independent *t*-test was conducted to compare the level of realism for the VR and 360° video. There was no significant difference in the scores for the 360° video (*M* = 1.5, *SD* = 2.03) and VR (*M* = 1.95, *SD* = 1.46; *t* (34.5) = 0.80, *p* = 0.42, two-tailed). The magnitude of the differences in the means (mean difference = 0.45, 95% *CI*: −0.06 to 1.59) was small (*Cohen’s d* = 0.25).

While the level of presence did not present significant statistics, a Chi-square goodness-of-fit test indicates there was a significant statistical difference in the proportion of the participants with a preference for VR (85%) over the 360° video experience (15%) compared with a hypothesized even split between the technologies, χ^2^ (2, *N* = 20) = 9.8, *p* = 0.002.

After performing a few non-parametric statistics, we reached the conclusion that the independent *t*-test demonstrated similar results to other viable non-parametric statistics. One example of a non-parametric statistic performed was the Mann–Whitney U test, which revealed no significant difference in the level of presence of the 360° video (*M* = 2.20, *n* = 20) and VR (*M* = 2.80, *n* = 20), *U* = 266, *z =* 2.10, *p =* 0.07.

Parametric statistics can be used with Likert data, with small sample sizes, with unequal variances, and with non-normal distributions, with no fear of coming to the wrong conclusion [66].

### 4.2. Discussion

**Figure 6:**
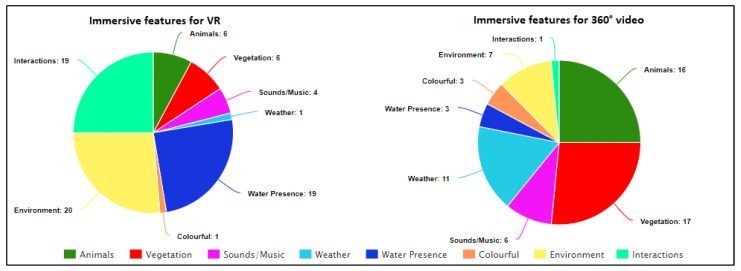
Immersive features highlighted by participants on both experiences.

The data presented above outline the opinions, points of view, preferences, and issues of older people in relation to the VR and 360° outdoors immersive experiences. As observed in question 21 of Table 1, there is a clear preference for the VR technology, and the data suggest that the ‘freedom to interact with the environment’ is the primary factor in participant choice. Interactivity was identified as a key factor for consideration in future work. An analysis of the videos identified several environmental features within both experiences (Figure 6) that participants found attractive, including: the use of surrounding sounds [37,38,39], natural environments, including colorful fauna and flora [14,40,67] (implicating the importance of nature), and relaxing music [8,22,61]. Almost all of the participants (95%) commented positively on the presence of water within the VR experience and suggested that they found the waves and associated sounds within the Driftwood experience extremely calming. This is evidenced by the comment ‘*My feet are wet!*’ being uttered as the participant raised her legs to avoid touching the virtual water.

Figure 6 presents a comparison between the 360 video and VR experiences in terms of the features that participants identified as contributing to the level of immersion. The comparison focuses primarily on the characteristics of the outdoor environments, such as the presence of water, weather, and flora and fauna, and not the interaction.

When the videos are analyzed, it can be tentatively suggested that the optimal experience for older people is an interactive VR experience that implements the immersive features, substantiating the findings of other studies [37,38], to provide an enjoyable nature-oriented immersive experience. However, several common design and user experience issues were raised by participants, and these include:

- **Control and navigation:** Learning the basic controls proved to be complex for some participants in the VR Driftwood experience. Guidance on how to navigate the environment and interact with virtual objects was commonly requested by the participants. The design of the intuitive navigation and interaction interfaces for older participants is an essential consideration for future VR experiences designed for the target demographic [24].
- **Dizziness/focus issues:** The participants frequently commented that the experiences resulted in dizziness and a loss of focus. This is possibly caused by technical limitations, such as the poor resolution and refresh rate of the headset [68], and from the use of masks and glasses [22,43]. The techniques used to minimize sickness during the trials included asking the participants to remain seated and limiting the time usage.
- **Weight issues:** The headset feeling heavy or uncomfortable was an issue comparable to other studies [22,37]. The participants remained in a seated position [11], which minimized the effects of the weight of the headset.
- **Bad perspective issues:** The perspective was not pleasant for the participant; this issue was exclusive to the 360° experience where the camera height was unrealistic, with a feeling like flying. To prevent this, ensuring that the camera is at head level is important to increase the sense of presence and realism, adapting to the common height of the participants.
- **Other issues:** These include difficulties in reaching the ground to pick up objects and suggestions for immersion improvement (pedals, bucket, and treadmill). Because the participants were aged citizens, there were physical restrictions that made it difficult for them to bend down and interact with the small objects on the ground.

The positive psychological effects observed were generally related to relaxation, such as the weather in the 360° video and the waves in the VR experience. The participants also reported feeling motivated to explore the possibilities of the technology, feeling happy and joyful during and after the trials. However, the participants also reported negative effects from experiences related to the frustration of not being able to do something (complexity of controller), complaints linked to dizziness issues, and the fear or worry of doing something wrong (mostly at the beginning of the session, because the longer they experienced the simulations, the lower the feelings of fear). Those negative effects can be addressed through a better UX design and onboarding participants.

The physiological reactions from the participants can be directly related to psychological improvements and to the technological immersive nature of VR. Major movements observed from the recordings include the participants putting their feet up to avoid getting wet with virtual water, demonstrating the immersive capabilities of the VR experience; this also justifies the greater sense of presence experienced in a VR environment compared with that of a 360° video experience in the questionnaire. Another major movement identified was the participants pointing at something, for example, a bird or vegetation, and laughing, which demonstrates fascination, curiosity, and joy.

Although the participants reported a greater sense of realism while engaged with the VR experience, possibly because of the degree of freedom to explore the environment, it was observed on the video analysis that when the participants were asked to move their feet to simulate walking, they experienced a more realistic simulation, and they felt present inside the virtual environment as if it was real.

### 4.3. Limitations of the Research

The data would suggest that VR is the preferred experience of the research participants; however, this is largely based on the degree of interactivity offered by the VR experience. Cognizance should be given to the absence of a longitudinal study on interactivity and the maintenance of the participant’s interest over time. In addition, there are ‘glitches’ in the 360°degree video experience, for example, the camera height and the weather conditions, that if compensated for in a follow-on video may change the outcomes. Interactivity can be built into a 360° video through layered augmented reality and route choice, although the participant will still follow a predefined route. VR experiences are expensive to produce, and require significant expertise, but generic models, an object-oriented approach, and gamified assets can help reduce the cost and complexity, providing users with an immersive experience that is close to the reality of nature. The restrictions on the time taken to interview participants and to conduct trials in an efficient manner limits the content of the questionnaire and the richness of the information that can be extracted; however, this limitation is compensated for by video captures of the participants undergoing the trial. Finally, the descriptive statistics used as the basis of this paper do not address statistical significance.

Due to a small data sample, it is difficult to generalize across studies or in a broader context; therefore, the results of the study and the conclusions made represent ‘potential trends’.

## 5. Conclusions

With the growth of the older population across Europe [44], the need for investments in improving the wellbeing of older people is paramount. Immersive technologies can provide a stimulating environment for older, more frail individuals, allowing them to experience an intuitive, meaningful simulation of a natural environment that contributes to their psychological and physiological wellbeing. This research outlines the technological preferences of older people in relation to immersion in natural environments created using VR and 360 video. The data collected indicate that the preference for VR is due to its ability to imbue participants with a sense of presence in the virtual world through the freedom of interaction, captivating older people for longer periods of time, implying that older people are more interested in the interaction provided by the technology rather than photorealism.

According to the data analysis and confirmed by the literature review, there are a number of factors that contribute to a meaningful virtual experience for older people, such as compatible and simplistic interactions [37,39] to ensure older people are able to independently use the technology; the use of sounds and relaxing music [33,37,38,39] for improved mental wellbeing and immersion; procedures to avoid cybersickness (seated position and limiting time) [11,14,22,37,39] to prevent unwanted health issues; and the freedom of choices [38,39,61] for greater enjoyment. In addition, in this paper, multiple issues have important implications for the development of future experiences, and these include:

- The resolution and loss-of-focus issues, minimized by the optimization of the software and hardware.
- An inconvenient camera perspective, improved by recording from head height.
- The weight of the device and the complexity of the controllers, which can be addressed through simple user interfaces and a better UX design.

Future work will incorporate an assessment of the psychology and physiology of older participants before, during, and after technology sessions rather than simple observation. Experiences need to be designed to minimize the identified issues, adapt controllers for use by older people, and provide straightforward interfaces.

## Figures and Tables

**Table 1:** Comparison between questionnaire results for each technology using mean score.

Question	Range		Mean VR	Mean 360°
Q1. How aware were you about the real world during experience?	−3 = Extremely aware	+3 = Not at all	*M* = 2.50	*M* = 1.85
Q2. How real did the virtual world seem to you?	−3 = Not real at all	+3 = Completely real	*M* = 1.95	*M* = 1.50
Q3. Sensed like a virtual world instead of real environment.	−3 = Feels unreal	+3 = Feels real	*M* = 2.55	*M* = 1.35
Q4. How consistent is the virtual environment with a real world experience?	−3 = Inconsistent	+3 = Consistent	*M* = 1.80	*M* = 1.30
Q5. How real did the virtual world seem to you?	−3 = As real as an imagined world	+3 = Indistinguishable from the real world	*M* = 1.10	*M* = 1.00
Q6. Did you fell present in virtual space?	−3 = Not present	+3 = Present	*M* = 2.75	*M* = 2.00
Q7. I was not aware of my real environment.	−3 = Aware	+3 = Not aware	*M* = 2.30	*M* = 1.85
Q8. In a virtual world, I had a sense of “being there”.	−3 = Not present	+3 = Present	*M* = 2.80	*M* = 2.20
Q9. I felt like the virtual world surrounded me.	−3 = Not surrounded	+3 = Surrounded	*M* = 2.80	*M* = 2.50
Q10. I felt present in virtual space.	−3 = Not present	+3 = Present	*M* = 2.45	*M* = 2.00
Q11. I still paid attention to the real environment.	−3 = Paid attention	+3 = Did not pay attention	*M* = 1.75	*M* = 1.50
Q12. Virtual world seemed more realistic than the real world.	−3 = Real world more realistic	+3 = Virtual world more realistic	*M* = 0.75	*M* = 0.40
Q13. I felt like I was just perceiving pictures.	−3 = Fully disagree	+3 = Fully agree	*M* = −1.40	*M* = −1.15
Q14. I was completely captivated by the virtual world.	−3 = Not captivated	+3 = Captivated	*M* = 2.85	*M* = 2.00
Q15. I felt afraid in Virtual world.	−3 = Afraid	+3 = Not afraid	*M* = 2.80	*M* = 2.45
Q16. Experience captivated me.	−3 = Not captivated	+3 = Captivated	*M* = 2.95	*M* = 1.75
Q18. I did not feel comfortable.	−3 = Uncomfortable	+3 = Comfortable	*M* = 3.00	*M* = 1.80
Q19. I experienced pain or discomfort during trial.	−3 = Pain	+3 = No pain	*M* = 2.80	*M* = 2.74
Q20. I could not see or hear properly. *N* = 13	−3 = Problems	+3 = No problems	*M* = 2.31	*M* = 0.23
Q21. Which preference do you prefer? (*N*)	360° video	VR	VR (17)	Video (3)
Average immersion score	Sum of means by number of questions		2.15	1.55

## Data Availability

An anonymized dataset is available on request by emailing supervisor author—William Farrelly.
